# Supplementary material for: Resolving the full spectrum of human genome variation using Linked-Reads
Source: Genome Res. 2019 Apr;29(4):635–45. doi: 10.1101/gr.234443.118 (PMC6442396; doi:10.1101/gr.234443.118)
Supplement: Supplemental Material [file supp_gr.234443.118_Supplemental_longranger-2.2.zip › longranger-2.2.tar.bz2!/lr2.2-public/lib/go/src/code.google.com/vitess/third_party/py/mock-1.0.1.tar.gz!/mock-1.0.1/html/compare.html]

Mock Library Comparison — Mock 1.0.1 documentation


### Navigation

- index
- next |
- previous |
- Mock 1.0.1 documentation »

# Mock Library Comparison¶

A side-by-side comparison of how to accomplish some basic tasks with mock and
some other popular Python mocking libraries and frameworks.

These are:

- flexmock
- mox
- Mocker
- dingus
- fudge

Popular python mocking frameworks not yet represented here include
MiniMock.

pMock (last release 2004 and doesn’t import
in recent versions of Python) and
python-mock (last release 2005) are
intentionally omitted.

Note

A more up to date, and tested for all mock libraries (only the mock
examples on this page can be executed as doctests) version of this
comparison is maintained by Gary Bernhardt:

- Python Mock Library Comparison

This comparison is by no means complete, and also may not be fully idiomatic
for all the libraries represented. *Please* contribute corrections, missing
comparisons, or comparisons for additional libraries to the mock issue
tracker.

This comparison page was originally created by the Mox project and then extended for
flexmock and mock by
Herman Sheremetyev. Dingus examples written by Gary Bernhadt. fudge examples
provided by Kumar McMillan.

Note

The examples tasks here were originally created by Mox which is a mocking
*framework* rather than a library like mock. The tasks shown naturally
exemplify tasks that frameworks are good at and not the ones they make
harder. In particular you can take a Mock or MagicMock object and use
it in any way you want with no up-front configuration. The same is also
true for Dingus.

The examples for mock here assume version 0.7.0.

## Simple fake object¶

```
>>> # mock
>>> my_mock = mock.Mock()
>>> my_mock.some_method.return_value = "calculated value"
>>> my_mock.some_attribute = "value"
>>> assertEqual("calculated value", my_mock.some_method())
>>> assertEqual("value", my_mock.some_attribute)
```

```
# Flexmock
mock = flexmock(some_method=lambda: "calculated value", some_attribute="value")
assertEqual("calculated value", mock.some_method())
assertEqual("value", mock.some_attribute)

# Mox
mock = mox.MockAnything()
mock.some_method().AndReturn("calculated value")
mock.some_attribute = "value"
mox.Replay(mock)
assertEqual("calculated value", mock.some_method())
assertEqual("value", mock.some_attribute)

# Mocker
mock = mocker.mock()
mock.some_method()
mocker.result("calculated value")
mocker.replay()
mock.some_attribute = "value"
assertEqual("calculated value", mock.some_method())
assertEqual("value", mock.some_attribute)
```

```
>>> # Dingus
>>> my_dingus = dingus.Dingus(some_attribute="value",
...                           some_method__returns="calculated value")
>>> assertEqual("calculated value", my_dingus.some_method())
>>> assertEqual("value", my_dingus.some_attribute)
```

```
>>> # fudge
>>> my_fake = (fudge.Fake()
...            .provides('some_method')
...            .returns("calculated value")
...            .has_attr(some_attribute="value"))
...
>>> assertEqual("calculated value", my_fake.some_method())
>>> assertEqual("value", my_fake.some_attribute)
```

## Simple mock¶

```
>>> # mock
>>> my_mock = mock.Mock()
>>> my_mock.some_method.return_value = "value"
>>> assertEqual("value", my_mock.some_method())
>>> my_mock.some_method.assert_called_once_with()
```

```
# Flexmock
mock = flexmock()
mock.should_receive("some_method").and_return("value").once
assertEqual("value", mock.some_method())

# Mox
mock = mox.MockAnything()
mock.some_method().AndReturn("value")
mox.Replay(mock)
assertEqual("value", mock.some_method())
mox.Verify(mock)

# Mocker
mock = mocker.mock()
mock.some_method()
mocker.result("value")
mocker.replay()
assertEqual("value", mock.some_method())
mocker.verify()
```

```
>>> # Dingus
>>> my_dingus = dingus.Dingus(some_method__returns="value")
>>> assertEqual("value", my_dingus.some_method())
>>> assert my_dingus.some_method.calls().once()
```

```
>>> # fudge
>>> @fudge.test
... def test():
...     my_fake = (fudge.Fake()
...                .expects('some_method')
...                .returns("value")
...                .times_called(1))
...
>>> test()
Traceback (most recent call last):
...
AssertionError: fake:my_fake.some_method() was not called
```

## Creating partial mocks¶

```
>>> # mock
>>> SomeObject.some_method = mock.Mock(return_value='value')
>>> assertEqual("value", SomeObject.some_method())
```

```
# Flexmock
flexmock(SomeObject).should_receive("some_method").and_return('value')
assertEqual("value", mock.some_method())

# Mox
mock = mox.MockObject(SomeObject)
mock.some_method().AndReturn("value")
mox.Replay(mock)
assertEqual("value", mock.some_method())
mox.Verify(mock)

# Mocker
mock = mocker.mock(SomeObject)
mock.Get()
mocker.result("value")
mocker.replay()
assertEqual("value", mock.some_method())
mocker.verify()
```

```
>>> # Dingus
>>> object = SomeObject
>>> object.some_method = dingus.Dingus(return_value="value")
>>> assertEqual("value", object.some_method())
```

```
>>> # fudge
>>> fake = fudge.Fake().is_callable().returns("<fudge-value>")
>>> with fudge.patched_context(SomeObject, 'some_method', fake):
...     s = SomeObject()
...     assertEqual("<fudge-value>", s.some_method())
...
```

## Ensure calls are made in specific order¶

```
>>> # mock
>>> my_mock = mock.Mock(spec=SomeObject)
>>> my_mock.method1()
<Mock name='mock.method1()' id='...'>
>>> my_mock.method2()
<Mock name='mock.method2()' id='...'>
>>> assertEqual(my_mock.mock_calls, [call.method1(), call.method2()])
```

```
# Flexmock
mock = flexmock(SomeObject)
mock.should_receive('method1').once.ordered.and_return('first thing')
mock.should_receive('method2').once.ordered.and_return('second thing')

# Mox
mock = mox.MockObject(SomeObject)
mock.method1().AndReturn('first thing')
mock.method2().AndReturn('second thing')
mox.Replay(mock)
mox.Verify(mock)

# Mocker
mock = mocker.mock()
with mocker.order():
    mock.method1()
    mocker.result('first thing')
    mock.method2()
    mocker.result('second thing')
    mocker.replay()
    mocker.verify()
```

```
>>> # Dingus
>>> my_dingus = dingus.Dingus()
>>> my_dingus.method1()
<Dingus ...>
>>> my_dingus.method2()
<Dingus ...>
>>> assertEqual(['method1', 'method2'], [call.name for call in my_dingus.calls])
```

```
>>> # fudge
>>> @fudge.test
... def test():
...     my_fake = (fudge.Fake()
...                .remember_order()
...                .expects('method1')
...                .expects('method2'))
...     my_fake.method2()
...     my_fake.method1()
...
>>> test()
Traceback (most recent call last):
...
AssertionError: Call #1 was fake:my_fake.method2(); Expected: #1 fake:my_fake.method1(), #2 fake:my_fake.method2(), end
```

## Raising exceptions¶

```
>>> # mock
>>> my_mock = mock.Mock()
>>> my_mock.some_method.side_effect = SomeException("message")
>>> assertRaises(SomeException, my_mock.some_method)
```

```
# Flexmock
mock = flexmock()
mock.should_receive("some_method").and_raise(SomeException("message"))
assertRaises(SomeException, mock.some_method)

# Mox
mock = mox.MockAnything()
mock.some_method().AndRaise(SomeException("message"))
mox.Replay(mock)
assertRaises(SomeException, mock.some_method)
mox.Verify(mock)

# Mocker
mock = mocker.mock()
mock.some_method()
mocker.throw(SomeException("message"))
mocker.replay()
assertRaises(SomeException, mock.some_method)
mocker.verify()
```

```
>>> # Dingus
>>> my_dingus = dingus.Dingus()
>>> my_dingus.some_method = dingus.exception_raiser(SomeException)
>>> assertRaises(SomeException, my_dingus.some_method)
```

```
>>> # fudge
>>> my_fake = (fudge.Fake()
...            .is_callable()
...            .raises(SomeException("message")))
...
>>> my_fake()
Traceback (most recent call last):
...
SomeException: message
```

## Override new instances of a class¶

```
>>> # mock
>>> with mock.patch('somemodule.Someclass') as MockClass:
...     MockClass.return_value = some_other_object
...     assertEqual(some_other_object, somemodule.Someclass())
...
```

```
# Flexmock
flexmock(some_module.SomeClass, new_instances=some_other_object)
assertEqual(some_other_object, some_module.SomeClass())

# Mox
# (you will probably have mox.Mox() available as self.mox in a real test)
mox.Mox().StubOutWithMock(some_module, 'SomeClass', use_mock_anything=True)
some_module.SomeClass().AndReturn(some_other_object)
mox.ReplayAll()
assertEqual(some_other_object, some_module.SomeClass())

# Mocker
instance = mocker.mock()
klass = mocker.replace(SomeClass, spec=None)
klass('expected', 'args')
mocker.result(instance)
```

```
>>> # Dingus
>>> MockClass = dingus.Dingus(return_value=some_other_object)
>>> with dingus.patch('somemodule.SomeClass', MockClass):
...     assertEqual(some_other_object, somemodule.SomeClass())
...
```

```
>>> # fudge
>>> @fudge.patch('somemodule.SomeClass')
... def test(FakeClass):
...     FakeClass.is_callable().returns(some_other_object)
...     assertEqual(some_other_object, somemodule.SomeClass())
...
>>> test()
```

## Call the same method multiple times¶

Note

You don’t need to do *any* configuration to call mock.Mock() methods
multiple times. Attributes like call\_count, call\_args\_list and
method\_calls provide various different ways of making assertions about
how the mock was used.

```
>>> # mock
>>> my_mock = mock.Mock()
>>> my_mock.some_method()
<Mock name='mock.some_method()' id='...'>
>>> my_mock.some_method()
<Mock name='mock.some_method()' id='...'>
>>> assert my_mock.some_method.call_count >= 2
```

```
# Flexmock # (verifies that the method gets called at least twice)
flexmock(some_object).should_receive('some_method').at_least.twice

# Mox
# (does not support variable number of calls, so you need to create a new entry for each explicit call)
mock = mox.MockObject(some_object)
mock.some_method(mox.IgnoreArg(), mox.IgnoreArg())
mock.some_method(mox.IgnoreArg(), mox.IgnoreArg())
mox.Replay(mock)
mox.Verify(mock)

# Mocker
# (TODO)
```

```
>>> # Dingus
>>> my_dingus = dingus.Dingus()
>>> my_dingus.some_method()
<Dingus ...>
>>> my_dingus.some_method()
<Dingus ...>
>>> assert len(my_dingus.calls('some_method')) == 2
```

```
>>> # fudge
>>> @fudge.test
... def test():
...     my_fake = fudge.Fake().expects('some_method').times_called(2)
...     my_fake.some_method()
...
>>> test()
Traceback (most recent call last):
...
AssertionError: fake:my_fake.some_method() was called 1 time(s). Expected 2.
```

## Mock chained methods¶

```
>>> # mock
>>> my_mock = mock.Mock()
>>> method3 = my_mock.method1.return_value.method2.return_value.method3
>>> method3.return_value = 'some value'
>>> assertEqual('some value', my_mock.method1().method2().method3(1, 2))
>>> method3.assert_called_once_with(1, 2)
```

```
# Flexmock
# (intermediate method calls are automatically assigned to temporary fake objects
# and can be called with any arguments)
flexmock(some_object).should_receive(
    'method1.method2.method3'
).with_args(arg1, arg2).and_return('some value')
assertEqual('some_value', some_object.method1().method2().method3(arg1, arg2))
```

```
# Mox
mock = mox.MockObject(some_object)
mock2 = mox.MockAnything()
mock3 = mox.MockAnything()
mock.method1().AndReturn(mock1)
mock2.method2().AndReturn(mock2)
mock3.method3(arg1, arg2).AndReturn('some_value')
self.mox.ReplayAll()
assertEqual("some_value", some_object.method1().method2().method3(arg1, arg2))
self.mox.VerifyAll()

# Mocker
# (TODO)
```

```
>>> # Dingus
>>> my_dingus = dingus.Dingus()
>>> method3 = my_dingus.method1.return_value.method2.return_value.method3
>>> method3.return_value = 'some value'
>>> assertEqual('some value', my_dingus.method1().method2().method3(1, 2))
>>> assert method3.calls('()', 1, 2).once()
```

```
>>> # fudge
>>> @fudge.test
... def test():
...     my_fake = fudge.Fake()
...     (my_fake
...      .expects('method1')
...      .returns_fake()
...      .expects('method2')
...      .returns_fake()
...      .expects('method3')
...      .with_args(1, 2)
...      .returns('some value'))
...     assertEqual('some value', my_fake.method1().method2().method3(1, 2))
...
>>> test()
```

## Mocking a context manager¶

Examples for mock, Dingus and fudge only (so far):

```
>>> # mock
>>> my_mock = mock.MagicMock()
>>> with my_mock:
...     pass
...
>>> my_mock.__enter__.assert_called_with()
>>> my_mock.__exit__.assert_called_with(None, None, None)
```

```
>>> # Dingus (nothing special here; all dinguses are "magic mocks")
>>> my_dingus = dingus.Dingus()
>>> with my_dingus:
...     pass
...
>>> assert my_dingus.__enter__.calls()
>>> assert my_dingus.__exit__.calls('()', None, None, None)
```

```
>>> # fudge
>>> my_fake = fudge.Fake().provides('__enter__').provides('__exit__')
>>> with my_fake:
...     pass
...
```

## Mocking the builtin open used as a context manager¶

Example for mock only (so far):

```
>>> # mock
>>> my_mock = mock.MagicMock()
>>> with mock.patch('__builtin__.open', my_mock):
...     manager = my_mock.return_value.__enter__.return_value
...     manager.read.return_value = 'some data'
...     with open('foo') as h:
...         data = h.read()
...
>>> data
'some data'
>>> my_mock.assert_called_once_with('foo')
```

*or*:

```
>>> # mock
>>> with mock.patch('__builtin__.open') as my_mock:
...     my_mock.return_value.__enter__ = lambda s: s
...     my_mock.return_value.__exit__ = mock.Mock()
...     my_mock.return_value.read.return_value = 'some data'
...     with open('foo') as h:
...         data = h.read()
...
>>> data
'some data'
>>> my_mock.assert_called_once_with('foo')
```

```
>>> # Dingus
>>> my_dingus = dingus.Dingus()
>>> with dingus.patch('__builtin__.open', my_dingus):
...     file_ = open.return_value.__enter__.return_value
...     file_.read.return_value = 'some data'
...     with open('foo') as h:
...         data = f.read()
...
>>> data
'some data'
>>> assert my_dingus.calls('()', 'foo').once()
```

```
>>> # fudge
>>> from contextlib import contextmanager
>>> from StringIO import StringIO
>>> @contextmanager
... def fake_file(filename):
...     yield StringIO('sekrets')
...
>>> with fudge.patch('__builtin__.open') as fake_open:
...     fake_open.is_callable().calls(fake_file)
...     with open('/etc/password') as f:
...         data = f.read()
...
fake:__builtin__.open
>>> data
'sekrets'
```

### Table Of Contents

- Mock Library Comparison
  - Simple fake object
  - Simple mock
  - Creating partial mocks
  - Ensure calls are made in specific order
  - Raising exceptions
  - Override new instances of a class
  - Call the same method multiple times
  - Mock chained methods
  - Mocking a context manager
  - Mocking the builtin open used as a context manager

#### Previous topic

Further Examples

#### Next topic

CHANGELOG

### This Page

- Show Source

### Quick search


Enter search terms or a module, class or function name.

### Navigation

- index
- next |
- previous |
- Mock 1.0.1 documentation »

© Copyright 2007-2012, Michael Foord & the mock team.
Last updated on Nov 05, 2012.
Created using Sphinx 1.1.3.
